# A comprehensive study on the relieving effect of *Lilium brownii* on the intestinal flora and metabolic disorder in *p*-chlorphenylalanine induced insomnia rats

**DOI:** 10.1080/13880209.2021.2019283

**Published:** 2022-01-03

**Authors:** Yanpo Si, Wenjun Wei, Xiaohui Chen, Xiaolong Xie, Tao Guo, Yohei Sasaki, Youbo Zhang, Lili Wang, Fei Zhang, Shuying Feng

**Affiliations:** a Department of Pharmacy, Henan University of Chinese Medicine, Zhengzhou, China; b Graduate School of Medical Sciences, Kanazawa University, Kanazawa City, Japan; c State Key Laboratory of Natural and Biomimetic Drugs and Department of Natural Medicines, School of Pharmaceutical Sciences, Peking University, Beijing, China; d Medical College, Henan University of Chinese Medicine, Zhengzhou, China

**Keywords:** 5-Hydroxytryptamine, metabolic profiles, tryptophan metabolism, arachidonic acid metabolism, trimethylamine-*N*-oxide, kynurenic acid

## Abstract

**Context:**

The bulb of *Lilium brownii* F. E. Brown (Liliaceae) (LB) is a common Chinese medicine to relieve insomnia.

**Objective:**

To investigate the molecular mechanism of LB relieving insomnia.

**Materials and methods:**

Insomnia model was induced by intraperitoneally injection *p*-chlorophenylalanine (PCPA) in Wistar rats. Rats were divided into three groups: Control, PCPA (400 mg/kg, i.p. 2 days), LB (598.64 mg/kg, oral 7 days). The levels of 5-hydroxytryptamine (5-HT), norepinephrine (NE), melatonin (MT), and the expression of GABA_A_, 5-HT1A and MT receptors, as well as pathological changes in hypothalamus, were evaluated. 16S rDNA sequencing and UPLC-MS/MS were used to reveal the change of the intestinal flora and metabolic profile.

**Results:**

The adverse changes in the abundance and diversity of intestinal flora and faecal metabolic phenotype altered by PCPA in rats were reversed after LB treatment, accompanied by the up-regulated levels of 5-HT as 8.14 ng/mL, MT as 16.16 pg/mL, 5-HT1A R and GABA_A_ R, down-regulated level of NE as 0.47 ng/mL, and the improvement of pathological phenomena of cells in the hypothalamus. And the arachidonic acid metabolism and tryptophan metabolism pathway most significantly altered by PCPA were markedly regulated by LB. Besides, it was also found that LB reduced the levels of kynurenic acid related to psychiatric disorders and trimethylamine-*N*-oxide associated with cardiovascular disease.

**Conclusion:**

The mechanism of LB relieving insomnia involves regulating flora and metabolites to resemble the control group. As a medicinal and edible herb, LB could be considered for development as a health-care food to relieve increasing insomniacs in the future.

## Introduction

Insomnia is a frequent problem worldwide that affects people of all ages, with a major incidence in the elderly (Nau et al. 2005). It is generally accompanied by a short overall sleep duration, defined as difficulty falling asleep, repeated awakenings, or poor sleep quality (Doghramji 2006). The survey shows that 30% of the total global population is affected by mild insomnia and about 10% suffer from severe or chronic problems (National Institutes of Health 2005; Zhang et al. 2009). In this respect, insomnia is the second most common symptom after pain, which leads people to seek medical help (Mahowald and Schenck 2005). The reasons and mechanisms of insomnia are complex and can be symptoms of other major medical illnesses and psychiatric disorders, as well as the use, abuse, or exposure to certain substances (Wang et al. 2019). Insomnia is a popular and high recurrence mental disease, characterised by a variety of pathological changes. Simultaneously, a growing number of studies prove that gut microbiota interferes with gastrointestinal physiology, metabolism, and immune function of the host, and influences central nervous system (CNS) function and behaviour via the microbiota-gut-brain (MGB) axis (Foster and McVey Neufeld 2013; Luna and Foster 2015; Li et al. 2018). The delicate imbalance of gut microbiota is implicated in numerous human diseases, such as sleep loss, circadian misalignment, affective disorders, and metabolic disease (Agus et al. 2018).

The gut microbiota generally refers to the microorganisms that inhabit the gastrointestinal tract, approximately 1,000 types of microbiotas are present in the adult intestinal tract (Ley et al. 2006; Agus et al. 2018). Data from a study showed that a ‘reference man’ weighing 70 kg has 3.9 × 10^13^ bacteria in the gut, with the ratio of bacteria to human cells as 1:1 (Sender et al. 2016). In another study, it was reported that there is an average of 762,655 gut bacterial genes in each faecal sample, indicating the number of genes was 38 times that of the human genome (Brunkwall and Orho-Melander 2017). The above-mentioned numbers highlight the importance of the gut flora metabolic capacity, which is predicted to be even higher than that of the host. The effects of intestinal flora on the host’s physiological functions, such as metabolism, nutritional homeostasis, and immune system, as well as brain activity, are mediated by direct intercellular interaction and by metabolites that are either produced by the microbes or derived from the transformation of environmental or host molecules (Agus et al. 2018). Numerous studies have identified that gut microbiota affects brain function through a variety of pathways that generate a bidirectional communication of information (Macpherson and Harris 2004; Diaz Heijtz et al. 2011; Cryan and Dinan 2012; Breit et al. 2018). For example, the immunoregulatory pathway that the microbiota interacts with immune cells, affects the levels of cytokines and prostaglandin E2 (Feng et al. 2018), thereby affecting brain function. Another example is neuroendocrine pathways; the intestine, with more than 20 types of enteroendocrine cells, is the largest endocrine organ in the human body, producing molecules that can interact with the host physiology and initiate reactions at the local and distant levels (Zhang and Davies 2016). The gut microbiota can affect the hypothalamus-pituitary-adrenal (HPA) axis and CNS by regulating the secretion of neurotransmitters such as cortisol, tryptophan and 5-hydroxytryptamine (5-HT) (Raybould 2010). Moreover, the gut microbiota is also associated with insufficient sleep, circadian misalignment, affective disorders, and metabolic disease (Li et al. 2018). More recent research revealed that circadian clock misalignment and sleep deprivation changes circadian clock gene expression and microbial community structure (Davies et al. 2014; Johnston et al. 2016; Kunze et al. 2016; Wu et al. 2018). It was found that mice fed with *Lactobacillus rhamnosus* showed descending behaviours associated with anxiety and depression and changed cerebral expression of both γ-aminobutyrate (GABA) type A and GABA type B receptors compared with mice in the control group (Bravo et al. 2011). However, no such neurochemical and behavioural effects were observed in the vagal-severed animals with vagus nerve amputation, indicating that the vagus nerve is the main communication pathway between gut bacteria and the brain (Schulze et al. 2014).

Metabolomics has been used to investigate the characteristic changes in low-molecular-weight metabolites (Xu et al. 2016). This method offers a comprehensive view of the changes in a lot of metabolic and signalling pathways and their interactions (Iannucci et al. 2017). Because the changes in metabolite concentrations are more substantial and clearer than other ‘omics’ such as genomics, transcriptomics, and proteomics, metabolomics is more reliable, sensitive, and capable to reflect changes in biological function caused by disease or drug action (He et al. 2017; Li et al. 2018). Moreover, metabolomics is accepted to comprehensively evaluate endogenous metabolites of a biological system holistically, and its property is consistent with the holistic efficacy of Chinese medicine (Yang et al. 2012).

The use of Chinese herbal medicine for insomnia can be traced back to more than 2000 years ago (Shao et al. 2020). Many Chinese herbs that mainly include tonics, tranquilisers and tonic heat-clearing drugs have been widely used to treat insomnia (Song 2004). *Lilium brownii* F. E. Brown (Liliaceae) (LB) is a common medicinal species, the plant in Longhui County is famous as a geographical indication product in China. According to local official data, the planting area of LB in Longhui County alone reaches 2000 hm^2^ in 2020. The bulbs of LB known as ‘Bai-he’, have been widely used in Chinese folk medicine as a herb to remedy insomnia. Bai-he Lianzi soup, Gouqi Bai-he soup, and other soups prepared with LB as the main raw material are used in Chinese folks to relieve insomnia. In clinical practice, the Bai-he, a tranquilising agent, is used directly as Chinese medicine to treat insomnia and dreamful sleep by herbalist doctors and recorded in Pharmacopoeia of the People’s Republic of China (Munafo and Gianfagna 2015).

LB has been clinically used to treat insomnia for a long time, but its mechanism remains unexplored. In this research, an insomnia rats model induced by intraperitoneal (i.p.) PCPA was adopted. Related neurotransmitters, corresponding receptors, and pathology in the hypothalamus of insomnia rats were detected, and then an integrated approach of 16S ribosomal RNA (16S rRNA) gene sequencing combined with ultra-performance liquid chromatography-mass/mass spectrometry (UPLC-MS/MS) was applied to analyse the effects of LB on gut microbiota and faecal metabolic phenotype in PCPA-induced insomnia rats.

## Materials and methods

### Experimental instruments and reagents

UPLC-ESI-MS/MS system (MS, QTRAP® System, UPLC, Shim-pack UFLC SHIMADZU CBM30A system). UPLC: Waters ACQUITY UPLC HSS T3 C18 (1.8 µm, 2.1 mm × 100 mm); Ultra-High-Performance Liquid Chromatography (Dionex, USA); High-speed refrigerated centrifuge (Eppendorf, Germany); −80 °C Low-temperature freezer (Haier Company, China); A200 Gene Amplifier (Hangzhou Lange Scientific Instruments Co., Ltd., China); DL2000 DNA Maker (Nanjing, China); Stool DNA Kit (200) (Omega Bio-Tek, USA); AxyPrep polymerase chain reaction (PCR) Clean-up Kit (Axygen, USA); Qubit dsDNA HS Assay Kit (Invitrogen, USA); *p*‑chlorophenylalanine (C6506) (Sigma, USA).

### Experimental medication

Mature bulbs of LB were provided in March 2019 from the Longhui County of Shaoyang City, Hunan Province. The plant material was authenticated by Prof. Tao Guo (Henan University of Chinese Medicine). A voucher specimen with reference number 20190313 A was deposited in BN912 in Henan University of Chinese medicine. The fresh bulbs were peeled and extracted thrice with 60% ethanol [solvent/sample ratio 8:1 (v/w)] at room temperature for 14, 14 and 21 d, respectively, and then concentrated to obtain a brown extract with the yield of 10.69% (w/w).

### Experimental animals

The method was used according to the previously described technique by our team (Si et al. 2021). The specific pathogen-free (SPF) male Wistar rats (160−180 g) were purchased from Vital River Laboratory Animal Technology Co., Ltd. with licence number SCXK 2016-0006 (Beijing, China). The animals were kept at required room temperature (25 ± 2 °C) and relative humidity (50 ± 5%) with a diurnal cycle of 12 h and were given free access to water and food. All studies were approved by the Animal Experiments and Experimental Animal Management Committee from the Henan University of Chinese Medicine (Ethic approval document NO. DWLL202003253, Zhengzhou, China). The experimental procedures were carried out following the Animal Ethics Committee of the Henan University of Chinese Medicine.

## Rat sample collection

After acclimatisation for one week, the rats were randomly divided into three groups with eight rats per group: the control group (received saline only), PCPA group (400 mg/kg PCPA, Sigma No: C6506), LB group (400 mg/kg of PCPA + 598.64 mg/kg of LB). The rats in two groups involving PCPA received 400 mg/kg of PCPA suspended in saline (PH 7–8) by i.p. for two consecutive days. Rats in the control group were injected intraperitoneally at the same volume of a weakly alkaline saline solution only. All rats were supplied with either LB or an equal volume of water by oral administration for 7 consecutive days, respectively.

After 7 days of LB intervention, at least 5 faecal pellets from each rat were collected on ice in separate sterile centrifuge tubes and stored in liquid nitrogen without enzymes. After that, the faeces were immediately transferred and stored in a −80 °C freezer for further metabolic profiling and microbial community analysis, respectively.

### Assay for hypothalamic 5-HT, NE and MT

At the end of the experiment, the blood of rats was collected from the abdominal aorta. The whole-brain of each rat was immediately removed on ice after the head of the rats were cut off, and then the hypothalamus was quickly separated. The supernatant of hypothalamus tissue was obtained by homogenising and centrifuging the hypothalamus tissue at 5000 *g* for 10 min, the levels of 5-HT, melatonin (MT) and norepinephrine (NE) were determined by Enzyme-linked immunosorbent assay (ELISA) kit (Elabscience Biotechnology Co., Ltd., China).

### Histological observation

The hypothalamic lesions were assessed by haematoxylin and eosin (H&E) staining. The hypothalamus collected from rats were fasted overnight, fixed in 10% paraformaldehyde for 12 h at 4 °C, dehydrated with a series of 50–100% ethanol solutions, clarified with xylene, and embedded with paraffin. Tissue slices with a thickness of 5 µm were stained with haematoxylin and eosin and observed under the 200 × light microscopy to evaluate the pathological changes.

### Western blot analysis

Western blot analyses of hypothalamic rat samples were performed using radioimmunoprecipitation assay buffer (Servicebio Biotechnology, China). The protein samples (40 μg for each) were separated on a 10% polyacrylamide SDS-PAGE gel, transferred onto a polyvinylidene difluoride membrane (0.22 μm, Millipore, USA), and blocked with 5% skimmed milk at 37 °C for 1 h, followed by immunostaining with primary antibodies, and then incubated overnight at 4 °C and washed with TBST for 3 times. Subsequently, the membranes were incubated at room temperature for 1 h with appropriate secondary horseradish peroxidase (HRP)-conjugated antibodies. The band intensities were measured with the Alpha Innotech alphaEaseFC luminescent image analyser.

### UPLC-MS/MS analysis

The thawed faecal pellets (50 mg) were homogenised with 500 μL of ice-cold methanol/water (containing 1 μg/mL of 2-chlorophenylalanine). The mixtures were added cold steel balls to homogenate at 30 Hz for 3 min and then centrifuged for 10 min with 12,000 rpm at 4 °C. The supernatant collected was analysed *via* the data acquisition instrument system of UPLC-ESI-MS/MS. The liquid phase conditions were as follows: chromatographic column as Waters ACQUITY UPLC HSS T3 C18; mobile phase A as ultrapure water with 0.04% acetic acid and mobile phase B was acetonitrile with 0.04% acetic acid; elution gradient, 95:5 V/V at 0 min, 5:95 V/V at 11.0 min, 5:95 V/V at 12.0 min, 95:5 V/V at 12.1 min, 95:5 V/V at 14.0 min; column temperature as 40 °C; flow rate as 0.4 mL/min; injection volume as 2 μL. LIT and triple quadrupole scans were gained on a triple quadrupole-linear ion trap mass spectrometer (QTRAP), QTRAP^®^ LC-MS/MS System, operating using both positive and negative ion modes.

### 16 s rDNA microbial community analysis

Total genomic DNA from different faeces samples was extracted using the DNA Kit (E.Z.N.A.V ®Stool, D4015, Omega, Inc., USA). The V3-V4 variable region of the 16S rRNA gene was amplified with primers 341 F (5′-CCTACGGGNGGCWGCAG-3′) and 805 R (5′-GACTACHVGGGTATCTAATCC-3′). The 5′ ends of the primers were labelled with specific barcodes per sample and generic sequencing primers. PCR amplification was carried out with a total volume of 25 μL reaction mixture including 25 ng of template DNA, 12.5 μL of PCR Premix, 2.5 μL of each primer and PCR-grade water regulated volume. The PCR products were confirmed with 2% agarose gel electrophoresis. We purified the PCR products using AMPure XT beads (Beckman Coulter Genomics, Danvers, MA, USA) and analysed them using Qubit (Invitrogen, USA). The amplicon pools were used for sequencing, the size of the amplicon library was evaluated on Agilent 2100 Bioanalyzer, and the quantity of the amplicon library was analysed by using the Library Quantification Kit for Illumina (Kapa Biosciences, USA).

### Statistical analysis

Statistical analysis was performed with Statistical Product and Service Solutions (SPSS) 25.0. Statistical significance among the groups was conducted by One-Way ANOVA, and quantitative data were represented as means ± standard deviation. *p* < 0.05 were considered statistically significant. The raw data obtained *via* UPLC-MS/MS was processed by Profile Analysis software and then input to the SIMCA-P13.0 software for principal component analysis (PCA) and orthogonal partial least squares discriminant analysis (OPLS-DA) to obtain the score map. For identification of potential markers, those with the parameter with *p*-value <0.05 and variable importance in projection (VIP) value >1.0 among the three groups were included, following databases including Kyoto Encyclopaedia of Genes and Genomes (KEGG), Human Metabolome Database (HMDB) and METLIN were used. The discriminant molecules were input into the MetaboAnalyst for metabolic pathway analysis and network analysis was performed using Metscape 3.1. All samples were sequenced on an Illumina NovaSeq platform following the instructions of the manufacturer.

## Results

### Effects of LB on body weight of PCPA-induced insomnia rats

**Figure 1.**
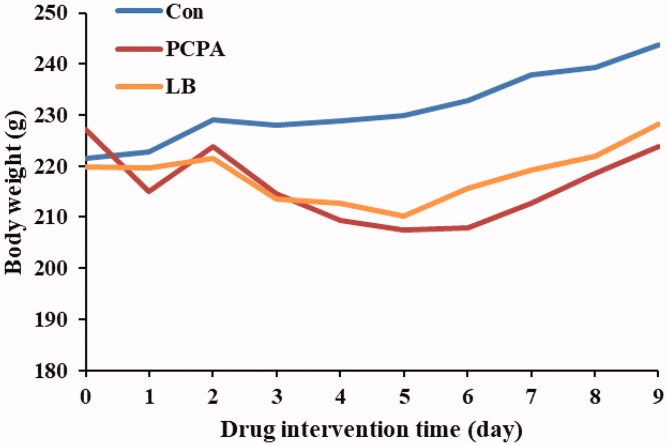
Effect of LB on body weights in PCPA-induced insomnia rat. The bodyweight of the animals is weighed for 10 consecutive days. Animals were intraperitoneally injected PCPA for the first two days and administered by gavage for 7 days.

The results showed that there was a difference in the body weight change in rats between the control group and the PCPA group (Figure 1). Compared with the PCPA group, a significant increase in body weight gain was noted in the LB group.

### Effects of LB on 5-HT, NE and MT of the hypothalamus

**Figure 2.**
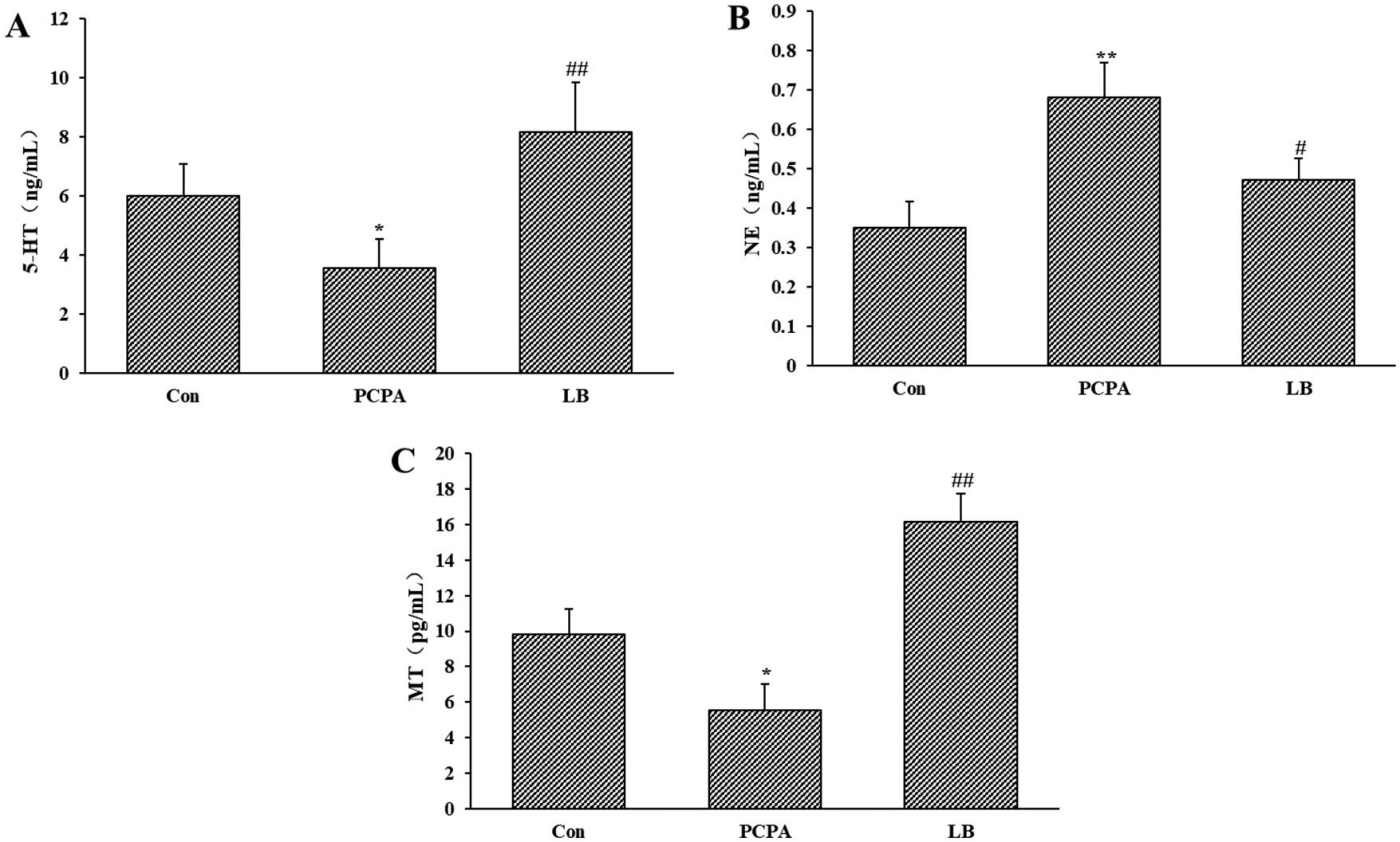
Effects of LB on hypothalamic levels of 5-HT (A), NE (B) and MT (C) in PCPA-induced insomnia rat. Control: received only saline treatment; PCPA: treated with 400 mg/kg PCPA only; LB: PCPA + 598.64 mg/kg LB. Values are presented as the means ± SD, *n* = 8. *p < .05, **p < .01 compared with Control; ^#^*p* < .05, ^##^*p* < .01 compared with PCPA using One-Way ANOVA.

As shown in Figure 2(A–C), PCPA administration dramatically decreased the 5-HT and MT levels (*p* < 0.05) of the hypothalamus and increased the level of NE (*p* < 0.01). Oral administration of LB (598.64 mg/kg) daily for 7 days produced a remarkable (*p* < 0.01) increase in 5-HT and MT levels and a decrease in NE level.

### Histopathological observation

**Figure 3.**
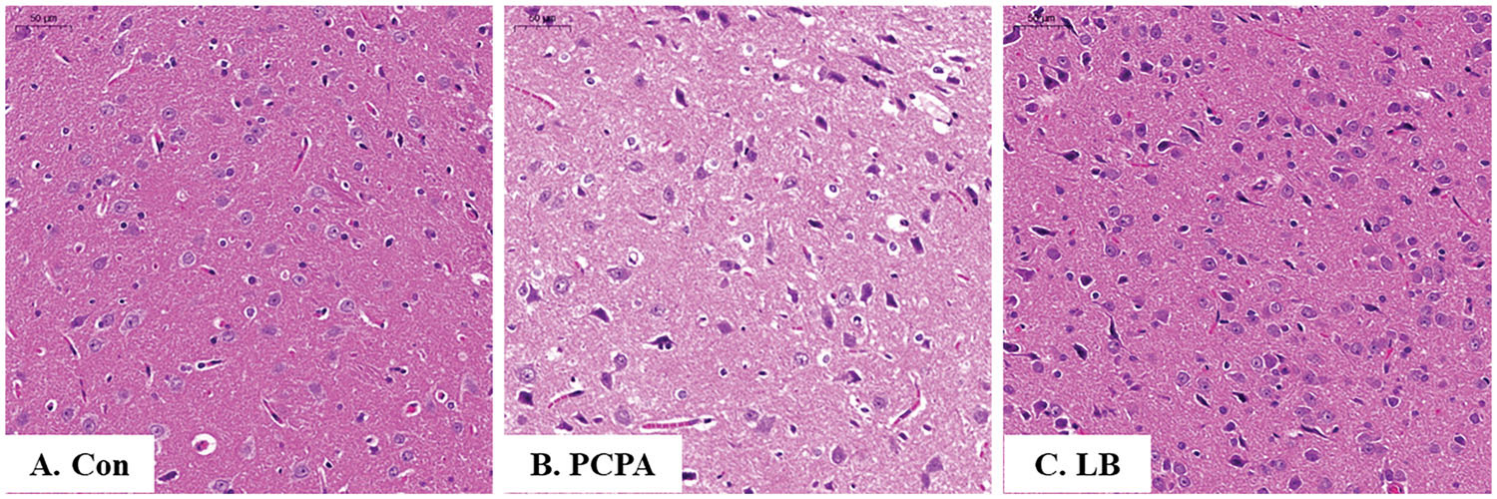
Histopathological observation of hypothalamic slices in rats. Hypothalamic sections were stained with haematoxylin and eosin (H&E stain 200×). (A) Con: Control (untreated); (B) PCPA (400 mg/kg alone); (C) LB: PCPA + 598.64 mg/kg LB.

As shown in Figure 3(A–C), the nerve cells of the hypothalamus in the control group were not only abundant and in good shape, but also were evenly distributed, and visible, while those in the PCPA group were severely deformed, not arranged tightly, and there are many broken small cells. Notably, in the LB group, the cells were arranged neatly, and the number of atrophy cells was significantly reduced.

### Effects of LB on the expression of GABA_A_ R, 5-HT1A and MT of hypothalamic

**Figure 4.**
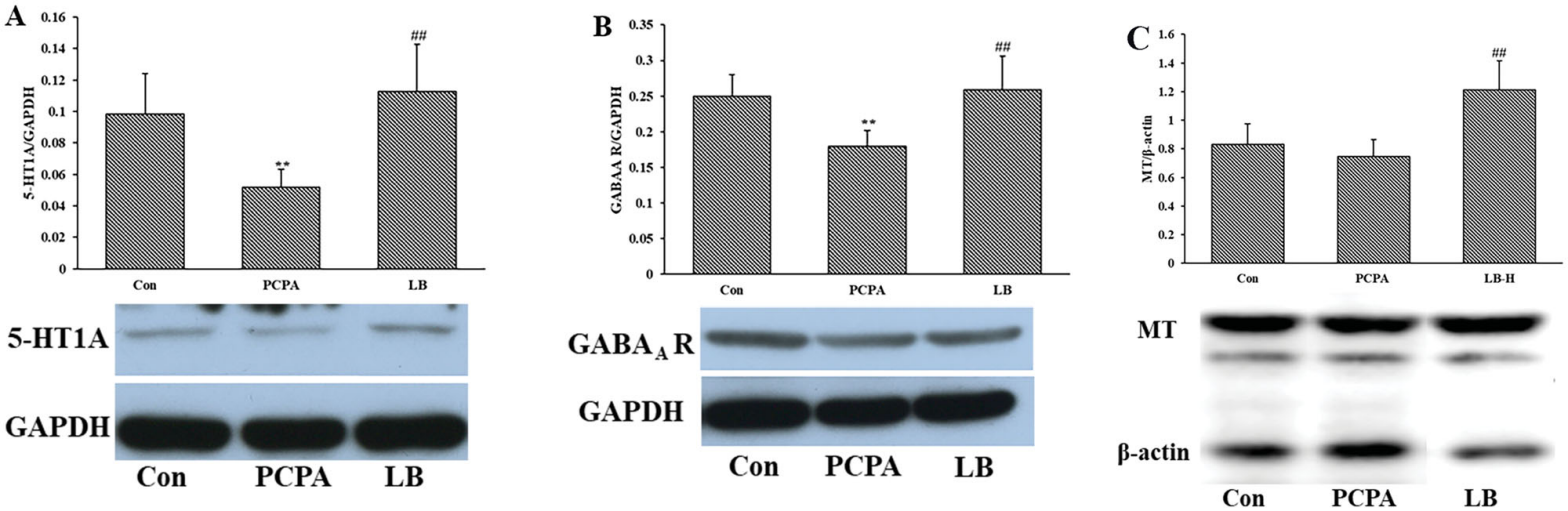
Effects of LB on the hypothalamic levels of 5-HT (A), NE (B) and MT (C) in PCPA-induced insomnia rats. Control: untreated; PCPA: 400 mg/kg PCPA alone; LB: PCPA + 598.64 mg/kg LB. Values are presented as the means ± SD, *n* = 8. Different letters indicate significant differences (*p* < .05) among samples by One-Way ANOVA test.

Shown as in Figure 4(A–C), PCPA treatment significantly lessened the expression of 5-HT and GABA_A_ R (*p* < 0.01) of the hypothalamic but did not significantly change the expression of MT. Oral administration of LB (598.64 mg/kg) daily over 7 days generated a significant increase (*p* < 0.01) in the expression of 5-HT1A, GABA_A_ R and MT.

### Bacterial community composition in faeces

**Figure 5.**
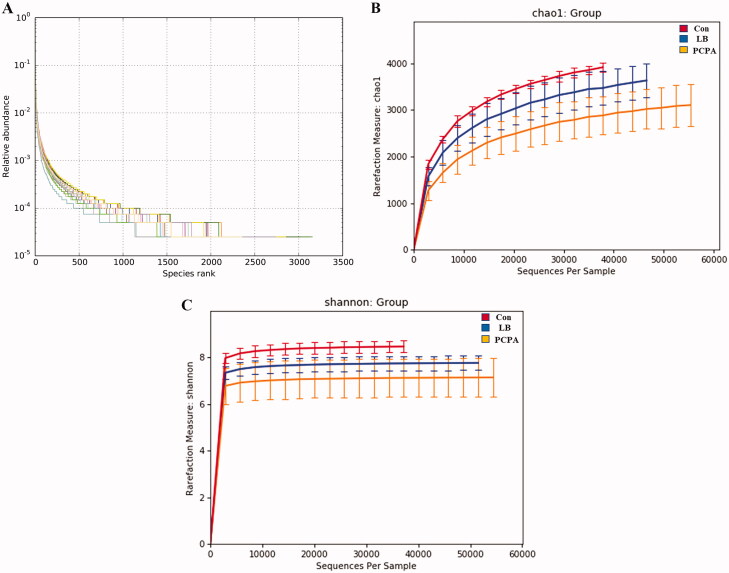
Changes in the richness and diversity of intestinal flora caused by LB treatment. (A) The OTU rank abundance of the intestinal flora in rats. (B) Bacterial richness estimated based on the Chao 1 value; (C) Bacterial diversity estimated from the Shannon index. The red, blue and yellow curves represent the control, LB and PCPA groups, respectively.

We sequenced the V3-V4 variable region of the 16S rRNA gene. 1,014,588 sequences have remained after effective reads were selected. Based on 97% sequence similarity cut-off point, the high-quality sequences were clustered into 51,342 operational taxonomic units (OTUs). In the LB group, the slope of the OTU rank abundance on the horizontal axis was gentler and the distribution was wider, indicating that the intestinal flora was more diversified and distributed more evenly after LB addition (Figure 5(A)). Simultaneously, the rarefaction curves approaching a plateau indicated that the sequencing depth contained rare new phylotypes and most of the diversity (Figure 5(B,C)). The estimated value of Chao1 reflects the ecological species richness of the bacterial community and protrudes species numbers, while the Shannon index positively correlates with the species distribution. The Chao1 index with 3539.91 vs. 2901.54 of average values (*p* < 0.01) (Figure 5(B)) and the Shannon index with 7.84 vs. 7.11 of average values (*p* < 0.05) (Figure 5(C)) in the LB group were higher than those in PCPA group.

### Effect of LB on the intestinal flora of insomnia rats induced by PCPA

**Figure 6.**
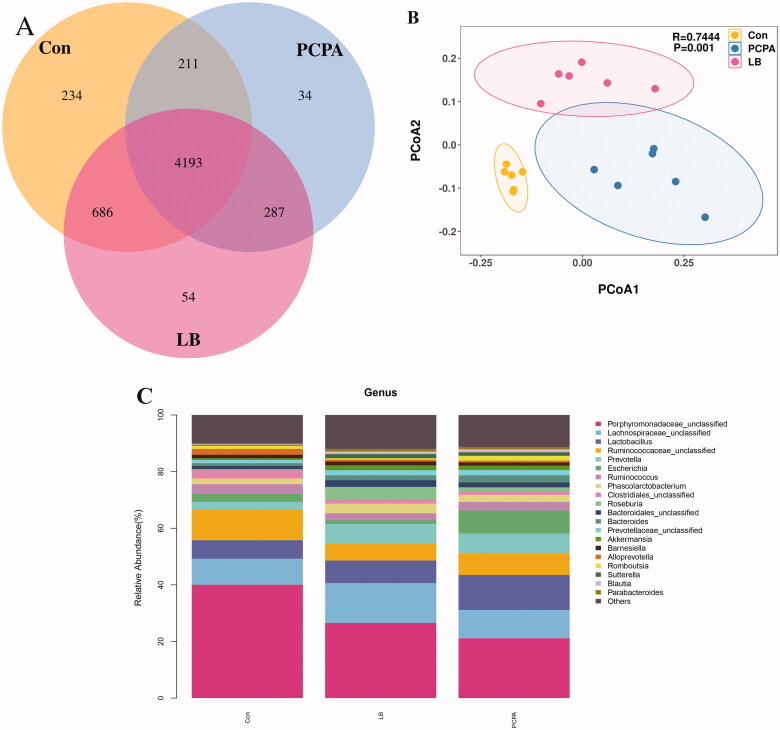
Effects of LB on intestinal flora in rats with insomnia induced by PCPA. (A) Veen diagram: The value inside the OTU in this area represents the number of OTUs. The control, PCPA and LB groups were marked yellow, blue and red, respectively. (B) PCoA score plots of OTUs for the control, PCPA and LB groups. (C) Relative gut microbiota abundance at the and genus-level in control, PCPA, and LB groups.

At the OTU level, the differences, and similarities between two groups of samples were counted, and common or unique OTUs among different samples were represented by the Venn diagram (Figure 6(A)). Compared with decreased diversity of the flora in the PCPA group (4725), the diversity of the flora increased significantly (5220) and tended to the normal group (5324) after the addition of LB. The principal coordinate analysis (PCoA) analysis showed that the community composition in the LB group was separated from the PCPA group (Figure 6(B)).

The taxon summary disclosed a noticeable change in the gut microbial composition after LB intervention. At the genus level, the relative abundance of taxa affected by LB included Porphyromonadaceae, Lachnospiraceae, *Lactobacillus*, Ruminococcaceae, *Prevotella*, *Escherichia*, *Ruminococcus*, *Phascolarctobacterium*, Clostridiales, *Roseburia,* Bacteroidales, *Bacteroides*, Prevotellaceae, *Akkermansia*, *Barnesiella*, *Alloprevotella*, *Romboutsia*, *Sutterella*, *Blautia*, *Parabacteroides*. The 10 floras at the genus level showed significant differences between the PCPA group and the control group (Figure 6(C)). Compared to the control group, the percentages of Porphyromonadaceae (18.92%, *p* < 0.01), Ruminococcaceae (6.46%, *p* < 0.01), Clostridiales (1.35%, *p* < 0.05) in the PCPA group were markedly decreased while the proportions of *Lactobacillus* (14.02%, *p* < 0.01), *Prevotella* (7.85%, *p* < 0.05), *Escherichia* (8.15%, *p* < 0.01), *Bacteroides* (1.81%, *p* < 0.01), and *Sutterella* (1.26%, *p* < 0.05)*, Blautia* (1.06%, *p* < 0.05)*, Parabacteroides* (0.81%, *p* < 0.05) were markedly increased. Simultaneously, the 5 flora at the genus level showed significant differences between the LB group and the PCPA group (Figure 6(C)). Compared to the PCPA group, the percentages of Porphyromonadaceae (28.83%, *p* < 0.05), Lachnospiraceae (14.06%, *p* < 0.05), and *Roseburia* (4.26%, *p* < 0.01) in the LB group were markedly increased while the proportions of *Lactobacillus* (6.13%, *p* < 0.01) and *Escherichia* (1.27%, *p* < 0.01) were markedly decreased.

### Effect of LB on the faecal metabolomics profiling in PCPA-induced insomnia rats

**Figure 7.**
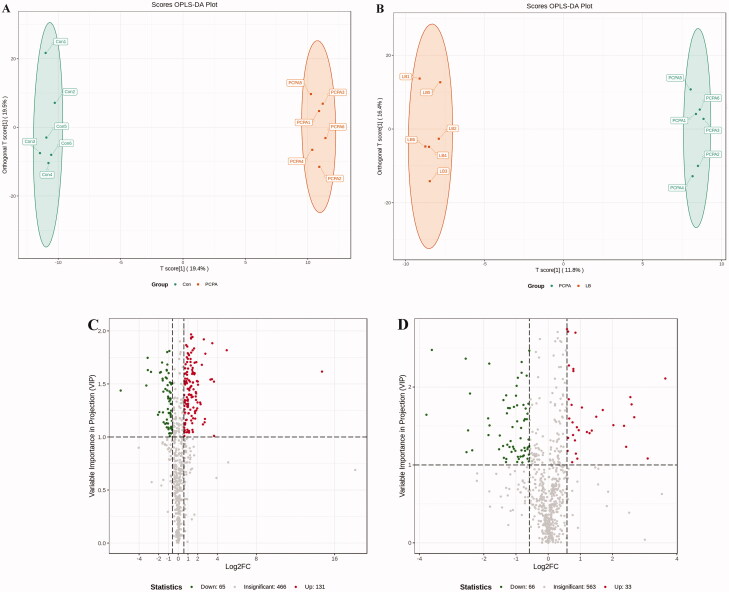
Effects of LB on the faecal metabolic profiles in PCPA-induced insomnia rats. (A, B) Score scatter plot of OPLS-DA in the PCPA and LB groups. Parameters for permutation test of OPLS-DA were *R*^2^*X* = 0.466, *R*^2^*Y* = 0.999 (*p* = 0.02), and *Q*^2^ = 0.657 (*p* = 0.025) in the PCPA group, *R*^2^*X* = 0.516, and *R*^2^Y = 0.998 (*p* = 0.03) and *Q*^2^ = 0.662 (*p* = 0.01) in the LB group. (C, D) Volcano plot: In the Vol-plot load graph, metabolite points that are significantly far from the origin (VIP > 1, *p* < 0.05) were selected as potential markers. Red dots represent upregulated metabolites, blue dots represent downregulated metabolites, grey dots indicate non-significant differences.

To further explore global metabolic alterations, faecal metabolic profiles in control, PCPA and LB groups were acquired *via* targeted metabolomics. To achieve a higher level of group separation and a better understanding of the classified variables, supervised OPLS-DA was carried out. The classified parameters indicate that this model has excellent performance on predictive ability (Figure 7(A,B)). According to the OPLS-DA model, the metabolic profile of the LB group was signally different from that of the PCPA group but tended to be like that of the control group, indicating that the bias caused by PCPA was markedly alleviated after LB addition. The values with VIP above 1.0 and *p*-values below 0.05 were selected for identifying significant metabolites related to group separation. The metabolites differentially expressed in the PCPA and LB groups were presented in the form of a volcano plot (Figure 7(C,D)). Variable value with VIP >1 was far away from the origin in the Volcano plot, which revealed the effects of potential biomarkers in different groups.

To identify differential metabolites between the PCPA and LB groups, we choose metabolites with fold change ≥ 1.5 (up-regulated) or ≤0.5 (down-regulated) in the PCPA and LB groups. These metabolites with VIP ≥ 1 were screened from the OPLS-DA model. Finally, 662 metabolites were screened from all samples (Figure 7(C,D)). Of these, 131 metabolites were upregulated, and 65 metabolites were downregulated after PCPA treatment (Figure 7(C)). Compared to the PCPA group, 33 metabolites were upregulated, and 65 metabolites were downregulated after LB administration (Figure 7(D)). These metabolites were categorised into more than 20 different classes, but the majority were organic acid and its derivatives, nucleotide and its metabolites, amino acid and its metabolites, oxidised lipid, carbohydrate and its metabolites, lipids others phospholipid, benzene and substituted derivatives, phenols and its derivatives, lipids, etc. As shown in Figure 7(E,F), the top 10 differential metabolites were up-regulated (marked in red) and down-regulated (marked in green) in the PCPA and LB groups. Among these total differential metabolites, a total of 37 metabolites were significantly down-regulated or up-regulated in the LB group compared with the PCPA group (Table 1).

**Table 1.** 37 Differential metabolites tending to normal levels after LB treatment.

Index	Metabolites	VIP	Class	Trend	
				LB group	PCPA group
MEDP831	1-Aminopropan-2-ol	1.73	Alcohol	down	up
MEDP039	Betaine	1.13	Alkaloid	down	up
MEDP214	Tryptamine	1.71	Amino acid and derivative	up	down
MEDN060	*N*-Isovaleroylglycine	1.38	Amino acid and derivative	up	down
MEDP084	Trimethylamine *N*-oxide	1.89	Amino acid and derivative	down	up
MEDP085	Tyramine	1.42	Benzene and derivative	up	down
MEDP819	Sulfadiazine	1.92	Benzene and derivative	down	up
MEDN670	*O*-Desmethylnaproxen	1.22	Benzene and derivative	down	up
MEDP205	L-Carnitine	1.79	Camitine	down	up
MEDN391	DHA [4Z,7*Z*,10*Z*,13*Z*,16*Z*,19*Z*-docosahexaenoic acid]	2.12	Lipid and derivative	down	up
MEDN751	(±)12-HETE [(±)12-hydroxy-5*Z*,8*Z*,10*E*,14*Z*-eicosatetraenoic acid]	1.77	Lipid and derivative	down	up
MEDN771	15-oxoETE [15-oxo-5*Z*,8*Z*,11*Z*,13*E*-eicosatetraenoic acid]	1.77	Lipid and derivative	down	up
MEDN765	11,12-EET [(±)11, (12)-epoxy-5*Z*,8*Z*,14*Z*-eicosatrienoic acid]	1.72	Lipid and derivative	down	up
MEDN763	(±)9-HETE [(±)-9-hydroxy-5*Z*,7*E*,11*Z*,14*Z*-eicosatetraenoic acid]	1.59	Lipid and derivative	down	up
MEDN782	8,9-EET [(±)8,9-epoxy-5*Z*,11*Z*,14*Z*-eicosatrienoic acid]	1.49	Lipid and derivative	down	up
MEDN557	*cis*-5-dodecenoic acid	1.11	Lipid and derivative	down	up
MEDN533	Xanthosine	2.02	Nucleotide and derivate	down	up
MEDN537	ADP-ribose	1.6	Nucleotide and derivate	down	up
MEDP148	1-Methylxanthine	1.38	Nucleotide and derivate	down	up
MEDP155	5-Methylcytosine	1.26	Nucleotide and derivate	down	up
MEDP430	2-Aminoadipic acid	1.87	Organic acid and derivative	up	down
MEDN339	Phenylpyruvic acid	1.62	Organic acid and derivative	up	down
MEDN581	Imidazoleacetic acid	1.5	Organic acid and derivative	up	down
MEDN472	1-Methyluric acid	2.3	Organic acid and derivative	down	up
MEDP069	*N*-Acetylputrescine	1.75	Organic acid and derivative	down	up
MEDP327	*N*-γ-Acetyl-*N*-2-formyl-5-Methoxykynurenamine	1.73	Organic acid and derivative	down	up
MEDN301	Caffeic acid	1.38	Organic acid and derivative	down	up
MEDN417	(RS)-Mevalonic acid	1.31	Organic acid and derivative	down	up
MEDP313	Guanidineacetic acid	1.24	Organic acid and derivative	down	up
MEDP319	Kynurenic acid	1.2	Organic acid and derivative	down	up
MEDP137	Protocatechuic aldehyde	1.19	Phenols and derivative	down	up
MEDP385	L-Sepiapterin	1.89	Pteridines and derivative	down	up
MEDP363	Biopterin	1.64	Pteridines and derivative	down	up
MEDP443	10-Formyltetrahydrofolate	1.44	Pteridines and derivative	down	up
MEDP249	Pantothenol	2.11	Vitamins and derivative	up	down
MEDP224	D-Fructose	2.18	Vitamins and derivative	down	up
MEDP244	All-*trans*-13,14-Dihydroretinol	1.08	Vitamins and derivative	down	up

Differential metabolites were identified using thresholds of VIP (variable importance in projection) ≥1.0 and fold change ≥1.5 (up-regulation) or ≤0.5 (down-regulation).

### KEGG classification and enrichment analysis of differential metabolites

**Figure 8.**
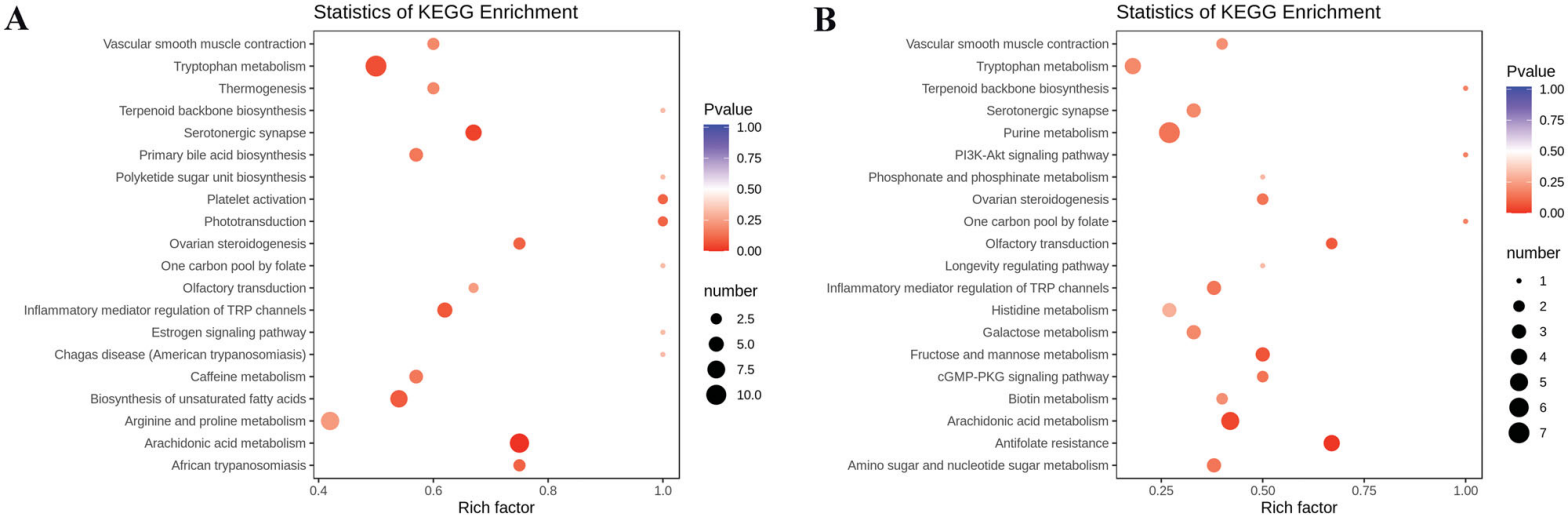
KEGG pathway enrichment analysis of differential metabolites after PCPA treatment (A) and LB treatment (B). The rich factor is the ratio of the number of differentially expressed metabolites in the corresponding pathway to the total number of metabolites annotated by this pathway. The larger the value, the greater the degree of enrichment. The colour of the dots is the *p*-value, the redder the more significant the enrichment. The size of the dots represents the number of differential metabolites enriched.

Subsequently, we carried out enrichment analysis for all differential metabolites using the KEGG. The research indicated the metabolites induced by PCPA were mainly associated with the arachidonic acid metabolism, tryptophan metabolism, primary bile acid biosynthesis, biosynthesis of unsaturated fatty acids, platelet activation, etc. (Figure 8(A)). Compared to the PCPA group, differential metabolites altered by LB were mainly associated with antifolate resistance, arachidonic acid metabolism, purine metabolism, tryptophan metabolism, Inflammatory mediator regulation of TRP channels, etc. (Figure 8(B)).

### Correlation analysis between the faecal microbiota and metabolites

**Figure 9.**
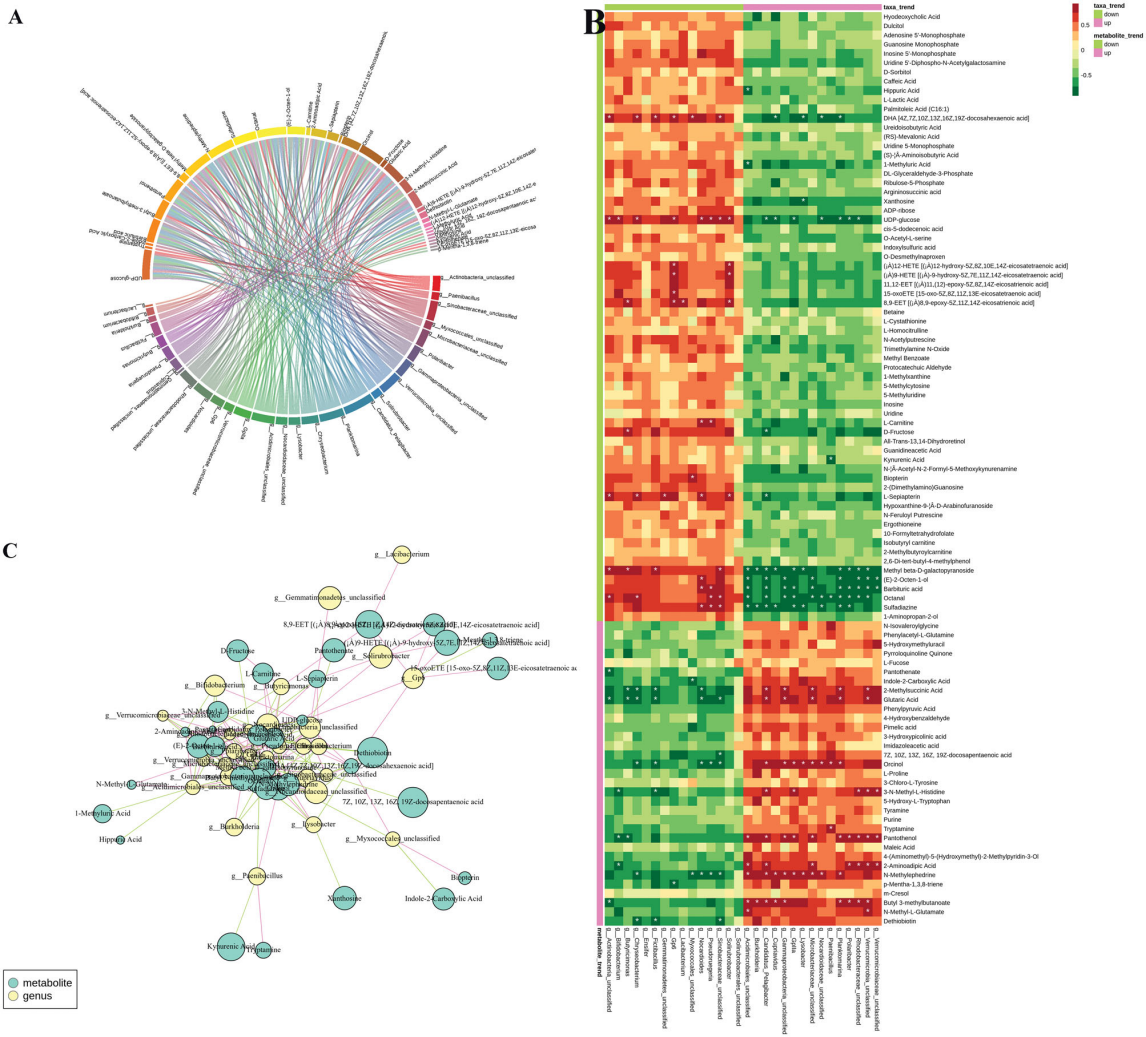
The relevance between the gut microbiota at the genus level and the differential faeces metabolites. (A) Chord diagram, the width of the chord link represents the strength of the interaction between the connected flora and the metabolites. The wider the link, the stronger the interaction. (B) Spearman’s correlation heat map: red indicates a positive correlation, while blue indicates a negative correlation. The deeper colour means a greater correlation (*p < 0.05). (C) The gut microbiota in genus level predicted by metabolic variation (*|r*| > 0.7, *p* < 0.05) is labelled with a similarity value. Lines connecting with metabolites (blue) show the direction of relevance to each genus of microbe (yellow) with the red (positive) or green (negative) lines.

To gain insight into whether the distribution of gut microbiota is related to the metabolic profiles, we analysed the correlation between significant different genus-level flora and differential metabolites using Spearman correlation analysis (|*r*| > 0.7). As shown in the heatmap (Figure 9) indicates that a total of 33 differential metabolites are positively or negatively correlated with one or more of 28 intestinal floras in the LB group. Among these, the metabolites that tend to be normal after LB treatment were (±)9-HETE, (±)12-HETE, 15-oxoETE, 1-methyluric acid, 2-aminoadipic acid, 8,9-EET, biopterin, d-fructose, DHA, kynurenic acid, l-carnitine, l-sepiapterin, pantothenol, sulfadiazine, tryptamine, xanthosine. 1-methyluric acid displayed a strong negative correlation with Acidimicrobiales. Sulfadiazine was negatively correlated with the *Lysobacter*, Nocardioidaceae, *Cupriavidus*, *Burkholderia*, Acidimicrobiales*, Candidatus, GPIIa, Planktomarina* and *Polaribacter*, and positively correlated with the *Nocardioides*, *Pseudoruegeria* and Sinobacteraceae. Tryptamine was positively correlated with *Paenibacillus*. Xanthosine was negatively correlated with *Lysobacter*. Pantothenol exhibited a positive correlation with Verrucomicrobia, Microbacteriaceae, γ-proteobacteria, Rhodobacteraceae, *Polaribacter*, *Candidatus*, *Planktomarina, GpIIa*, Acidimicrobiales and Verrucomicrobiaceae and a negative correlation with *Bifidobacterium* and *Butyricimonas*. Biopterin was positively correlated with Myxococcales. Kynurenic acid was negatively correlated with *Paenibacillus*. l-Carnitine was positively correlated with *Nocardioides* and *Pseudoruegeria*. 2-Aminoadipic acid was positively correlated with Acidimicrobiales, Verrucomicrobia, *Candidatus Pelagibacter,* Microbacteriaceae*, Polaribacter,* Rhodobacteraceae, Verrucomicrobia and Verrucomicrobiaceae and negatively correlated with *Bifidobacterium*. DHA was positively correlated with Actinobacteria, *Chryseobacterium*, *Fictibacillus*, Gp6, Myxococcales, sInobacteraceae and negatively correlated with *Cupriavidus*, *Lysobacter*, Nocardioidaceae, *Planktomarina*. D-Fructose was positively correlated with *Butyricimonas* and negatively correlated with *Candidatus pelagibacter*. l-Sepiapterin was positively correlated with Actinobacteria, *Chryseobacterium*, Gemmatimonadetes, *Nocardioides*, *Solirubrobacter* and negatively correlated with *Candidatus pelagibacter*. 15-oxoETE was positively correlated with Gp6. 8, 9-EET was positively correlated with *Solirubrobacter*, *Butyricimonas*, Gp6 and *Lacibacterium*.

## Discussion

Currently, the depletion of neurotransmitter 5-HT is considered one of the primary reasons for insomnia (De-Miguel and Trueta 2005). Studies revealed that the inhibition of 5-HT synthesis can generate the depletion of the brain 5-HT (Lepetit et al. 1991). One example of a 5-HT inhibitor is PCPA, which can selectively act on tryptophan hydroxylase (TpH) to inhibit enzyme activity and hinder 5-HT synthesis, leading to the disappearance of sleep circadian rhythm (Shi et al. 2018). The PCPA-induced rat model has been utilised to explore the ameliorative effect of Chinese medicine on insomnia. Therefore, in our study, the generation of the insomnia rat model was established through two consecutive injections of PCPA. After PCPA administration, daytime restlessness, insomnia, and weight loss were observed in rats (Figure 1(A)). A follow-up study showed that PCPA led to a prominent decrease in the 5-HT and MT levels (Figure 2(A,C)) as well as the expressions of 5-HT1A, GABA_A_ R and MT (Figure 4), and an increase in the NE level (Figure 2(B) in the hypothalamus. Besides, compared to the control group, the pathological observation of the hypothalamus of rats in the PCPA group showed that there were abundant broken cells and sparse neuronal cells (Figure 3(B)). These results indicated a successful establishment of the insomnia rat model by PCPA injection.

The results of the 16S rRNA gene sequencing demonstrated that there were significant differences in the abundance level and population diversity of intestinal microorganisms between the control group and PCPA group, as evidenced by the estimated Chao1 value, Shannon index, Venn diagram, PCoA diagram. The intervention of PCPA decreased the diversity of the microbiota and disturbed the homeostasis of intestinal flora (Figure 6(A–C)). The histogram results of species classification showed the flora at the genus levels had noteworthy differences between the PCPA and control group (Figure 6(C)). As shown in Figure 6(C), at the genus level, there are significant differences in 10 floras including Porphyromonadaceae, Ruminococcaceae, Clostridiales*, Lactobacillus*, *Prevotella*, *Escherichia, Bacteroides*, *Sutterella*, *Blautia,* and *Parabacteroides* between the PCPA and control groups. The results of OPLS-DA indicated there were significant differences in the distribution of faecal metabolites in the PCPA group compared with those of the control group (Figure 7(A)). In general, 196 metabolites were significantly changed. Of these, 131 and 65 metabolites were up-regulated and down-regulated in the PCPA group (Figure 7(C)), respectively. Further enrichment analysis of the differential metabolites using the KEGG revealed that the arachidonic acid metabolism and tryptophan metabolism are the most critical pathways involved in insomnia induced by PCPA in rats (Figure 8(A)).

Previously, it has been reported that LB could extend the sleep time of mice and shorten the sleep latency caused by pentobarbital sodium, which indicated that it had a better sedative and hypnotic effects (Wang et al. 2015). We found that LB could increase the body weight (Figure 1(A)) and the content of 5-HT and MT (Figure 2(A–C)) and reduce the level of NE (Figure 2(B)) in the hypothalamus of rats after LB administration. It was also observed that the pathology of the hypothalamus was significantly relieved (Figure 3(C)). Simultaneously, the results of 16S rDNA sequencing of intestinal flora showed that the values of Chao1 index (Figure 5(B)), Shannon index (Figure 5(C)) and OTUs (Figure 6(A)) in the LB group were higher than those in the PCPA group, indicating that the addition of LB could significantly increase the flora richness and diversity. As shown in Figure 6(B), the LB group was separated considerably from the PCPA group and clustered into a category with the control group, which indicated that the microbial species composition of the faeces in the LB group tends to be more similar to those in the control group. Furthermore, it was found that there are significant differences in the five floras, including Porphyromonadaceae, Lachnospiraceae, *Roseburia*, *Lactobacillus* and *Escherichia*, at the genus level between the LB and PCPA group (Figure 6(C)). Among the five floras, the Porphyromonadaceae, *Lactobacillus* and *Escherichia* in the genus level show a tendency of recovering from being those in a healthy control group after LB treatment. Collectively, our data suggest that the intervention of PCPA could significantly alter the gut microbial profile, and the addition of LB can correct substantially this bias. To the best of our knowledge, it was not reported that these bacteria including Porphyromonadaceae, *Lactobacillus* and *Escherichia*, are associated with insomnia in humans or animal models, which deserves further study.

The diversity and dynamics of the human gastrointestinal microbiome play a crucial role in the host health or disease (Tremaroli and Bäckhed 2012). The interrelationship between host and intestinal flora depends on complex molecular crosstalk, which is the basis of intestinal dynamic balance. A growing body of evidence manifests metabolites produced by gut microbiota as important mediators in host-microbial crosstalk. Similarly, metabolites generated by microbiota, including trimethylamine-*N*-oxide (TMAO) as a product of microbial-host co-metabolism of nutrients, betaine, and l-carnitine (Wang et al. 2011; Koeth et al. 2013), also affect host physiology. TMAO is produced by converting trimethylamine (TMA), which originates from bacteria, through the liver. Roberts et al. (2018) reported that the inhibitors that target the major microbial TMA-generating enzymes (CutC and CutD) could lower the levels of TMAO, prevent platelet activation, and hinder thrombus formation.

Some studies have shown that in human and animal studies the TMAO levels were closely associated with cardiovascular risks (Gao et al. 2014; Zhu et al. 2016). Studies have shown that l-carnitine levels of plasma in subjects experiencing cardiac evaluation and prediction raised risks for prevalent cardiovascular diseases (CVD) and incident major adverse cardiac events, but only among the subjects with concurrently high TMAO levels (Koeth et al. 2013). In our study, TMAO, betaine and l-carnitine in the PCPA group were also elevated, indicating that insomnia caused by PCPA injection may increase the risk of CVD. Furthermore, it was reported that subjects were classified into different enterotypes bade on faecal microbial composition. The subjects with an enterotype characterised by enrichment of *Prevotella* had higher TMAO concentrations of plasma than those with an enterotype characterised by enriched in proportions of *Bacteroides* (Koeth et al. 2013). It is worth noting that the abundance of *Prevotella* and *Bacteroides* at the genus level (Figure 3(E)) in the PCPA group was increased, accompanied by the higher level of TMAO. In addition, it was also reported that vegans or vegetarians with a higher proportion of *Lachnospira* in the intestinal have lower TAMO levels (Koeth et al. 2013). Although the abundance of *Prevotella* and *Bacteroides* did not change significantly after LB treatment, while the level of Lachnospiraceae increased significantly, indicating the regulation effect of LB did not completely reverse all change of the flora caused by PCPA. The reduction of TMAO level also showed that LB treatment could decrease the cardiovascular risk caused by PCPA-induced insomnia in rats.

Compared with the PCPA group, 33 and 66 metabolites were upregulated and downregulated in the LB group, respectively (Figure 7(D)). Among these differential metabolites, a total of 37 metabolites returned to a healthy level after LB intervention (Table 1). The KEGG pathway analysis of metabolites in the LB group showed that the tryptophan metabolic pathway, as well as arachidonic acid metabolic pathway highly associated with insomnia, were mainly adjusted (Figure 8(B)). Insomnia is known to cause disorders in tryptophan metabolism (Bhat et al. 2020). It can be seen from the results of differential metabolites that the tryptamine in the PCPA group was significantly reduced, while the *N*-γ-acetyl-*N*-2-formyl-5-methoxykynurenamine (AFMK), as the enzymatic degradation production of 5-HT, was elevated. To our knowledge, tryptamine, as a tryptophan catabolite produced by the gut microbiota, is a β-arylamine neurotransmitter, which is generated by l-tryptophan through the action of pyridoxal phosphate (PLP)-dependent decarboxylases (Williams et al. 2014). Tryptamine, structurally related to serotonin, can be a precursor of the neurotransmitter 5-HT and likely binds to the same site(s) (Mousseau and Butterworth 1994).

In the intestinal tract, tryptamine induces the release of the neurotransmitter 5-HT from enterochromaffin cells (ECs) (Takaki et al. 1985) located on the mucosal surfaces, and then 5-HT excites gastrointestinal movement by acting on neurons in the enteric nervous system (Mawe and Hoffman 2013). Under physiological conditions, more than 90% of 5-HT is generated in the gut, particularly in ECs, and the peripheral 5-HT does not cross the blood-brain barrier. But peripheral production of 5-HT by ECs is affected by the gut microbiota, even if 5-HT does not cross the blood-brain barrier, the central serotoninergic pathways may be influenced indirectly by the gut microbiota through regulating tryptophan and tryptamine availability (Agus et al. 2018). It is worth noting that the decrease of tryptamine is also consistent with the description ‘PCPA is an inhibitor of TpH, which can inhibit the synthesis of 5-HT belonging to tryptophan metabolism’ and our experimental results ‘the reduction of 5-HT in the hypothalamus. The metabolism of the tryptophan to 5-HT process is done by the tryptophan hydroxylase 1 enzyme (TpH1), producing 5-HT, which is further metabolised into 5-HT *via* aromatic amino acid decarboxylase. Subsequently, 5-HT is further metabolised to MT, which has a sedative and hypnotic effect and regulates the sleep-wake cycle (Golombek et al. 2015), AFMK is produced by MT catalysed by indoleamine 2, 3-dioxygenase (Chen et al. 2015). Notably, the level of MT in the hypothalamus of insomnia rats was reduced, while the AFMK, as a downstream metabolite of MT, was increased. Therefore, we speculated that the degradation of enzymes related to 5-HT metabolism was enhanced in PCPA-induced insomnia rats. After administration of LB, the level of 5-HT in the hypothalamus and the abnormal levels of differential metabolites, including tryptamine and AFMK, were significantly adjusted in faeces. Therefore, it is believed that LB significantly improves the degradation of enzymes related to 5-HT metabolism.

Furthermore, it can be found that after PCPA treatment, kynurenic acid (KA), as a secondary metabolite of tryptophan in the kynurenine pathway (KP), is significantly increased (Table 1). Currently, it was accepted that abnormal KP is involved in neurological disorders (Lovelace et al. 2017), CVD (Song et al. 2017) and stroke (Colpo et al. 2019). The view that enhancement of KA levels in CNS could underlie cognitive decline is supported by the increasing KA metabolism in Alzheimer’s disease, down’s syndrome, and the enhancement of KA function during the early stage of Huntington’s disease (Kepplinger et al. 2005). Moreover, the KA levels are higher in the cerebrospinal fluid and critical CNS regions of schizophrenics (Muller and Schwarz 2006). Therefore, it seems that PCPA-induced insomnia will also increase the risk of psychiatric disorders in rats. The level of KA was significantly reduced after LB administration. Notable, according to the results of correlation analysis between bacterial flora and metabolites, it was found that the decrease of KA was also correlated with the increase of *Paenibacillus* at the genera level (*p* < 0.05). Therefore, we speculated that *Paenibacillus* might be involved in KA metabolism in the KP pathway. Intestinal tryptophan metabolism is regulated directly or indirectly by the microbiota. Thus, from a therapeutic perspective, tryptophan metabolism in the gut is an operable actor, using molecules that target specific pathways or employing microorganisms that control tryptophan metabolism as probiotics.

After sleep deprivation, the 15-oxoETE (15-KETE) and 8, 9-EET in arachidonic acid metabolic pathways were significantly down-regulated and up-regulated, respectively. Both LB and Suanzaoren (*Semen ziziphi spinosae*), as a tranquiliser of Chinese medicine (Wang et al. 2012) could regulate the arachidonic acid metabolic pathway. After administration of LB, the two metabolites mentioned above were restored to healthy levels. However, the association between these two metabolites and insomnia remains unclear.

Similarly, differential metabolites involved in other metabolic pathways, such as imidazoleacetic acid (IMA), are also significantly regulated by LB. IMA is a naturally occurring metabolite that activates GABA_A_ receptors, and an analog of GABA where the amino group of GABA has been replaced by an imidazole ring (Johnston 2002). IMA may induce a state similar to natural sleep when injected intraperitoneally in the dose range of 400–500 mg/kg to the tested animals (Rick et al. 1973). It was also reported that IMA might non-competitively inhibit GABA-transaminase and increase the GABA level in cortical tissues (Rick et al. 1973). In our study, LB significantly recovered from the reduction in IMA levels induced by PCPA.

## Conclusions

LB may relieve insomnia through improving the disturbance of intestinal flora and metabolites caused by PCPA, and be considered for development as a health-care food to deal with increasing patients with insomnia.
